# Large sized bilateral adrenal myelolipoma with spontaneous retroperitoneal hemorrhage: a case series

**DOI:** 10.1097/MS9.0000000000003551

**Published:** 2025-07-18

**Authors:** Prasanna Subedi, Susmita Khadka Chhetri, Tek Nath Yogi, Amrit Bhusal, Nakendra Malla, Rumit Jha, Grishma Khadka, Pravakar Shrestha, Sarada Khadka

**Affiliations:** a Department of Surgery, BP Koirala Institute of Health Sciences (BPKIHS), Dharan, Sunsari, Nepal; b Department of Radiodiagnosis & Imaging, BP Koirala Institute of Health Sciences (BPKIHS), Dharan, Sunsari, Nepal; c Head of Department, Breast & Endocrine Surgery, BP Koirala Institute of Health Sciences (BPKIHS), Dharan, Sunsari, Nepal

**Keywords:** adrenal, incidentaloma, laparoscopy, myelolipoma

## Abstract

**Introduction:**

Adrenal myelolipomas are rare, benign adrenal tumors typically discovered incidentally. Histologically, it comprises mature adipose tissue and normal hematopoietic elements (erythroid, myeloid, and megakaryocytic precursors). Although often asymptomatic, larger tumors may present with mass effect, retroperitoneal hemorrhage, or spontaneous rupture requiring surgical intervention.

**Case Presentation:**

Both patients, a 51-year-old male and a 57-year-old female, presented with left-sided flank pain and imaging findings of large left adrenal masses (>10 cm) with retroperitoneal hemorrhage. Hormonal assays were negative, ruling out functional adrenal tumors. In both cases, laparoscopic adrenalectomy was attempted but converted to open surgery due to tumor size, friability, and intraoperative challenges. Bilateral adrenal myelolipoma and dense adhesion with the tail of the pancreas and splenic hilum was present in the first case.

**Discussion:**

Spontaneous rupture of large-sized adrenal myelolipoma occurs, primarily in tumors >10 cm, leading to hemorrhagic events. Imaging, particularly CT and MRI, aids diagnosis, revealing characteristic fat-rich lesions. Management depends on size, symptoms, and imaging findings. Tumors <10 cm are typically treated laparoscopically, while larger or invasive ones require open surgery. The prognosis is excellent, with minimal follow-up needed for most cases. For large tumors, periodic ultrasounds and clinical reviews are advised post-surgery.

**Conclusion:**

Most cases of adrenal myelolipomas are small, unilateral, and non-adherent to adjacent structures, but some may be bilateral, large, or adherent to nearby organs, leading to symptoms and intraoperative challenges. This case series emphasizes the complications associated with large adrenal myelolipomas and underscores the importance of careful evaluation, planning, and surgical approach.

## Introduction

Adrenal myelolipomas (AML) occur in 1 out of 500–1250 autopsy cases, comprising 0.08% to 0.2% of autopsies. Adrenocortical adenomas make up 60–70% of adrenal incidentalomas, with AML comprising 6–16% of cases, being the second most common adrenocortical incidentaloma. It was first marked out as “ad renal lipoma” by Arnoldin in 1866^[1]^, but the myeloid component was first described by Gierkein in 1905^[2]^. It is most commonly diagnosed between the fifth and seventh decades of life. AML is usually a benign, non-hormonal, and asymptomatic tumor. Histologically, it comprises mature adipose tissue and normal trilineage hematopoietic elements (erythroid, myeloid, and megakaryocytic precursors). In some cases, abdominal discomfort from an adrenal mass effect, overt hormone excess, or acute hemorrhage leads to identification^[3]^.

**HIGHLIGHTS**

- Adrenal myelolipomas are typically benign, non-hormonal, and asymptomatic tumors, often detected incidentally. However, they may present with symptoms such as abdominal discomfort, hormone excess, or acute hemorrhage.
- Spontaneous rupture is rare but more likely in large tumors, particularly those with high fat content. Larger tumors can lead to chronic hemorrhage or hemorrhagic shock.
- Treatment involves a multidisciplinary approach, with laparoscopic surgery preferred for tumors under 10 cm. Larger tumors or those with adhesions require open surgery.
- Both cases in this series were treated with open adrenalectomy after laparoscopic attempts were converted. In some cases, adrenal myelolipomas can adhere to surrounding structures like the spleen, aorta, and kidney, complicating surgery. Case 1 was bilateral and presented with splenic hilar vessel adhesion.

This case series has been reported in line with the PROCESS guidelines^[4]^.

## Case presentation

Both cases were selected via surgeon’s referral and were selected on the basis of similar nature and rarity.

### Case 1

A 51-year-old male patient with no history of alcohol use or smoking visited the Department of Surgical Gastroenterology of our hospital with a complaint of left flank pain 10 days prior. No h/o abdominal distension, fever, or trauma. He gave a history of being hypertensive for a few years but not under medication. At presentation, blood pressure was 150/100 mm of Hg and the abdomen was normally distended and nontender with no guarding.

**Figure 1.**
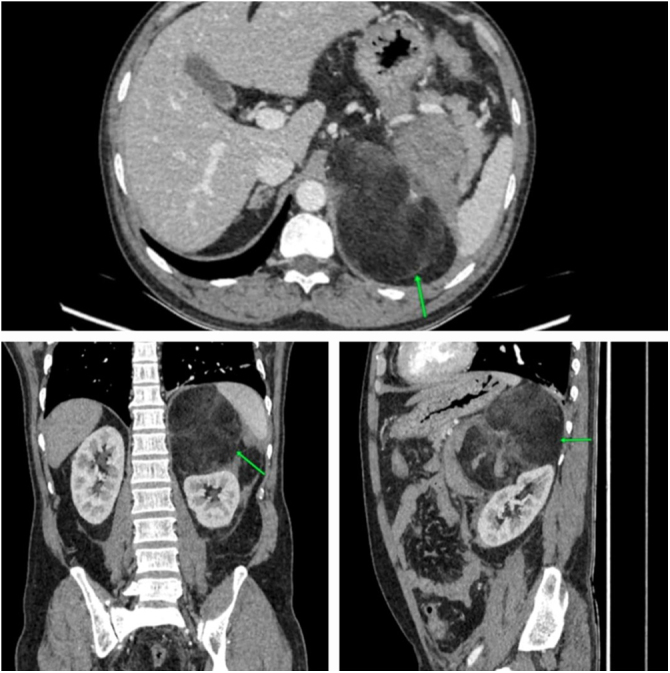
Case 1 – CT abdomen showing left adrenal myelolipoma (yellow arrow) of size 11*10*7.5 cm^3^ in anterior perirenal and pararenal space with non visualization of left adrenal gland separately, superolaterally abutting the spleen, inferiorly abutting and displacing left kidney, anteriorly abutting and displacing splenic vein and tail of pancreas,medially abutting the aorta and posteriorly abutting left hemidiaphragm.

**Figure 2.**
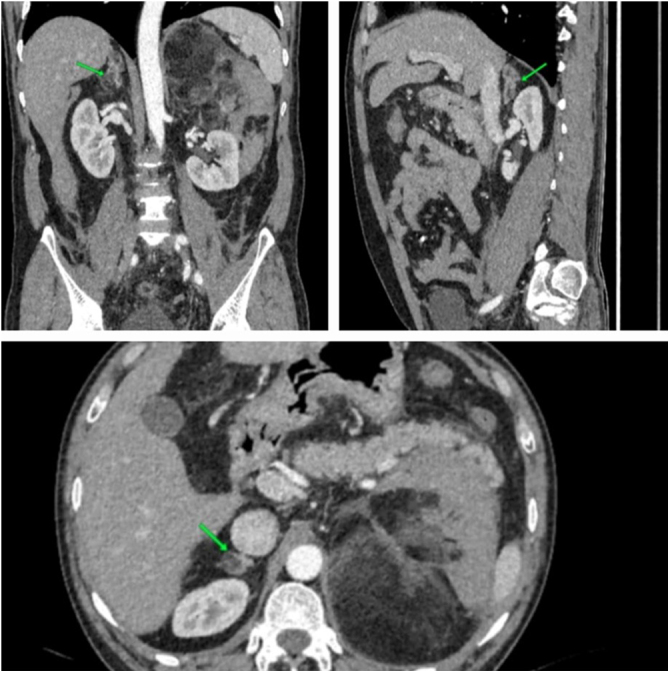
Case 1 – CT Abdomen showing the right adrenal gland with few predominantly fat-density lesions with intermixed soft tissue density suggesting adrenal myelolipoma (yellow arrow) largest measuring 14 mm*12 mm.

Besides cholelithiasis, computed tomography (CT) abdomen and pelvis showed a well-defined heterogeneous of size 11*10*7.5cc predominantly hypodense lesion of fat density (mean attenuation −127 HU) in left suprarenal region with anterior capsular defect with adjacent hyperdense area of blood density (mean attenuation ~66 HU) in anterior perirenal and pararenal space with non-visualization of left adrenal gland separately. The mass is anteriorly abutting and displacing the splenic vein and tail of the pancreas, medially abutting the aorta supero-laterally abutting the spleen, inferiorly abutting and displacing the left kidney and posteriorly abutting left hemidiaphragm (Fig. 1). On the right adrenal gland, few predominantly fat-density lesions with intermixed soft tissue density were noted largest measuring 14 mm*12 mm (Fig. 2). Mild high-density free fluid in the abdomen and pelvis suggestive of hemoperitoneum. Bilateral AML with spontaneous rupture of left AML with retroperitoneal hemorrhage was suspected based on these findings.

At the initial visit, his hemoglobin level was at the upper limit of normal, but in subsequent tests conducted 10 days later, it consistently remained at the lower limit of normal. Although the CT findings were consistent with AML, we assessed his hormone levels to rule out other hormonally active tumors, and all results were within normal limits.

**Figure 3.**
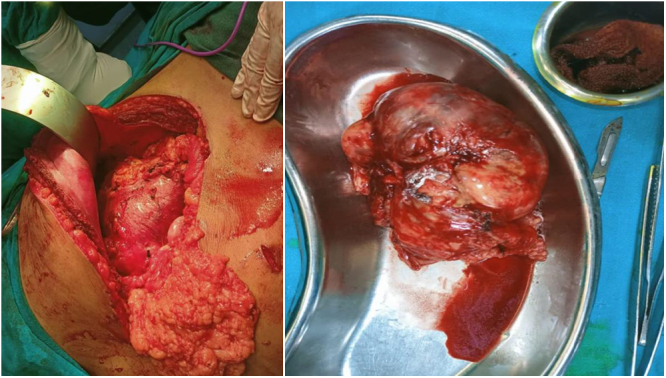
Case 1 – intraoperative view (left) and gross specimen (right) showing left adrenal myelolipoma of size 10 cm*12 cm.

He was then planned for laparoscopic/open left adrenalectomy with cholecystectomy surgery. In OT, initially, lap cholecystectomy was done then proceeded for adrenalectomy. The adrenal tumor was dissected from the surrounding structures but was densely adhered to the splenic hilar structures and pancreatic tail making it difficult to delineate separately. Massive bleeding occurred, so it was converted to open. Adrenal capsular hematoma ~20 cm was present separately from the adrenal tumor. Dense adhesions were present. An adrenal tumor of ~12*10 cm^2^ was taken out (Fig. 3). The splenic injury became apparent, so an open splenectomy was carried out. The right AML was not excised due to its small size and intraoperative complications.

**Figure 4.**
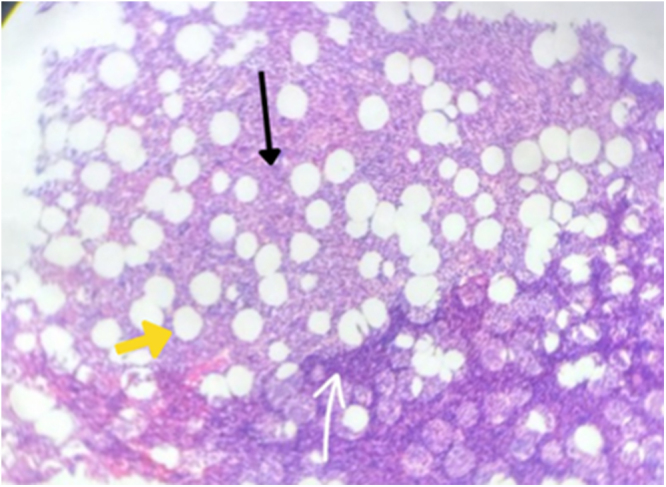
Case 1 histopathology (H&E) of left adrenal myelolipoma showing adrenal tissue (black arrow) and myeloid tissue (white arrow) interspersed with adipocytes (yellow arrow).

Histopathology of the adrenal mass showed fragments of circumscribed lesion comprising matured adipocytes admixed with aggregates of differentiating hematopoietic cells of three lineage predominantly erythroid and myeloid precursors along with increased megakaryocytes confirming myelolipoma (Fig. 4).

Gall bladder specimen showed features suggestive of chronic cholecystitis. Spleen specimen showed features suggestive of chronic venous congestion.

The patient was admitted to the ICU for 3 days. Drain fluid amylase was raised which made peri-operative pancreatic fistula (grade II) apparent. It was managed with IV fluid, antibiotics, and analgesics. Medicine consultation was done for post splenectomy vaccinations. The patient was discharged with stable hemodynamic parameters. The patient had an uneventful postoperative period, and no tumor recurrence was noted at 4 months follow-up. The patient was counseled about the right AML and for a yearly radiological evaluation.

### Case 2

A 57-year-old woman with a 6-year history of systemic hypertension, for which she was not on medication, presented to the surgery outpatient department with complaints of left flank and lumbar region pain persisting for 15 days. The pain was insidious in onset, dull-aching, constant, non-radiating, and mild to moderate in intensity, showing slight relief with analgesics and no identifiable aggravating factors. She denied any history of trauma, fever, nausea, vomiting, abdominal distension, weight loss, jaundice, changes in bowel habits, or urinary symptoms such as hematuria, dysuria, or increased frequency. Her history included cessation of alcohol consumption 12 years ago and a prior history of smoking. Physical examination revealed mild pallor, tachycardia, and a soft abdomen with mild tenderness over the left flank. Laboratory investigations showed mild anemia (Hb 9.1 g/dL).

**Figure 5.**
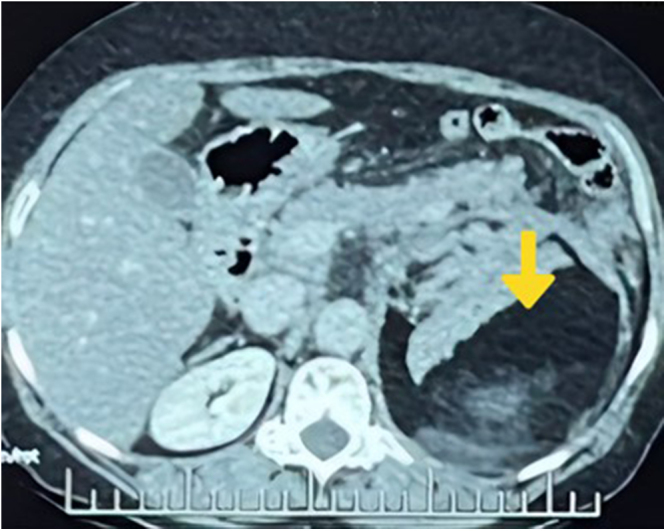
Case 2 – CT scan of the abdomen showing left adrenal myelolipoma (yellow arrow) measuring 11 cm × 10cm × 15 cm.

A contrast-enhanced CT scan of the abdomen and pelvis revealed a large, encapsulated, heterogeneous mass in the left suprarenal region measuring 11 × 10 × 15 cm, (Fig. 5) predominantly hypodense with areas of blood density, suggestive of a fat-containing lesion. The mass displaced adjacent structures, including the splenic vein and pancreatic tail anteriorly, the aorta medially, the splenic hilum superiorly, the left kidney inferiorly, and the posterior abdominal wall posteriorly. The left adrenal gland was not visualized, and there was thickening of the surrounding renal fascia with retroperitoneal fat stranding, raising suspicion of a ruptured AML. High-density free fluid in the pelvis suggested hemoperitoneum. Hormonal assays, including adrenocorticotropic hormone, cortisol, aldosterone, and epinephrine, were within normal limits. Differential diagnoses for the retroperitoneal fat-containing lesion included renal angiomyolipoma, liposarcoma, and lipoma.

**Figure 6.**
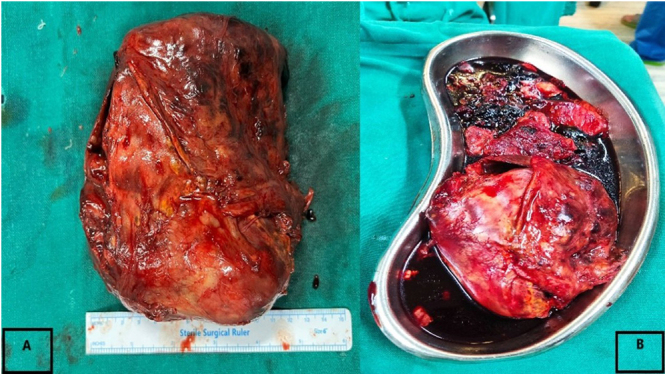
Case 2 – gross specimen of adrenal myelolipoma mass measuring 15 cm × 10 cm.

**Figure 7.**
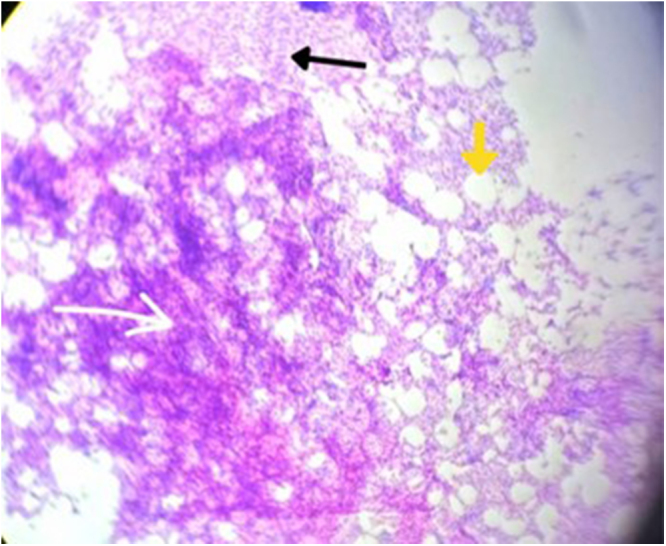
Case 2 – histopathology (H&E) of left adrenal myelolipoma showing adrenal tissue (black arrow) and myeloid tissue (white arrow) interspersed with adipocytes (yellow arrow).

Surgical management began with laparoscopic exploration, but due to the size and friability of the mass, the procedure was converted to open adrenalectomy via a Chevron incision. Intraoperative findings confirmed a large, ruptured adrenal mass measuring 15 × 10 cm at the superior pole of the left kidney, adherent to surrounding structures (Fig. 6). The mass was carefully excised, with a total weight of 450 g. Histopathological examination confirmed the diagnosis of AML (Fig. 7). The patient had an uneventful postoperative recovery, requiring no blood transfusion, and showed no evidence of tumor recurrence at an 8-month follow-up. The patient was counseled for a yearly radiological evaluation.

## Discussion

AML is typically a benign, non-hormonal, and asymptomatic tumor. In some cases, it may present with abdominal discomfort due to mass effect, overt hormone excess, or acute hemorrhage, leading to its diagnosis. While the disease is usually unilateral, it was bilateral in case 1. These tumors grow slowly, with an average growth rate of approximately 0.16 cm per year. Although reports of AML have significantly increased in recent decades, spontaneous rupture remains rare, occurring in approximately 4.5% of cases, with a median tumor size of about 120 mm, which can lead to chronic hemorrhage or, less commonly, hemorrhagic shock^[^5-8^]^. Larger lesions (typically >4 cm) and those with a high-fat content (>50%) are more prone to hemorrhagic events^[9]^.

Due to their high lipid content, myelolipomas exhibit characteristic imaging features. On pre-contrast CT, they show attenuation values below 0 HU, sometimes reaching below −50 HU. Adrenal nodules with more than 50% fat are strongly suggestive of myelolipoma^[10]^. While MRI is the most sensitive and specific modality for diagnosing adrenal hemorrhage, financial constraints in this case prevented its use.

The etiology of AML remains unclear. Proposed theories include metaplasia of reticuloendothelial cells in the adrenal glands, extramedullary hematopoiesis, ACTH stimulation, and interactions between mesenchymal stem cells and hematopoietic progenitors^[^11,12^]^.

Myelolipomas are usually well-defined tumors in the adrenal cortex, ranging in size from 4 to 6 cm, though larger tumors and even large ones up to 38 cm have been reported^[13]^. Spontaneous rupture is more likely in tumors exceeding 10 cm in diameter, especially those predominantly composed of fat.

Treatment decisions for AML require a multidisciplinary approach, factoring in symptoms, radiological findings, and patient-specific considerations. The traditional 4-cm threshold for surveillance is less relevant for lesions with characteristic myelolipoma imaging features and should not be the sole criterion for surgery^[14]^. Surgical options depend on tumor size and include laparoscopic or open techniques, such as transabdominal, lumbar, subcostal, or posterior laparotomy. Laparoscopic surgery is the preferred method for tumors smaller than 10 cm, provided they are well-defined and not invasive. For larger tumors (>10 cm) or those with significant adhesions or infiltration into surrounding structures, a midline open approach is recommended^[15]^.

AML generally have an excellent prognosis, and long-term follow-up is usually unnecessary. However, for large tumors, some studies recommend an abdominal ultrasound at 3–6 month intervals and annual clinical examinations post-surgery^[16]^. Transperitoneal laparoscopy has been shown to safely manage tumors exceeding 10 cm in diameter. While most cases proceed without requiring conversion to open surgery, isolated instances of conversion have been documented in the literature^[17]^.

Spontaneous rupture of AML is a rare but significant complication influenced by multiple histopathological factors. Previous studies have reported that increased vascularity with thin-walled, dilated blood vessels may predispose AMLs to hemorrhage, particularly in large tumors where rapid growth outpaces blood supply. Additionally, histopathological features such as intratumoral hemorrhage, necrosis, and granulomatous changes have been suggested to weaken tumor integrity, making it more susceptible to rupture under mechanical stress or increased internal pressure. Inflammatory responses may further contribute by increasing vascular permeability and friability. Larger AMLs (≥7 cm) have been associated with a higher risk of rupture due to vascular insufficiency and structural fragility, and comorbidities such as hypertension and obesity may exacerbate intra-abdominal pressure and vascular stress^[18]^. However, these findings were not noted in our case, as histopathological examination did not reveal significant vascular abnormalities, necrosis, or granulomatous inflammation. This suggests that factors beyond histopathology, such as mechanical compression from adjacent structures or sudden hemodynamic shifts, may also play a role in AML rupture.

Both of our cases presented with symptoms of mass effect like flank pain along with spontaneous retroperitoneal hemorrhage evident on CT along with anemia. Both cases had no abnormal hormone level, were in their 50s age group and had large left-sided AML which displaced/abutted adjacent vessels, organs like the aorta, spleen, kidney, etc. Both cases were initially approached laparoscopically due to the feasibility of minimally invasive adrenalectomy for large adrenal tumors. However, several intraoperative challenges necessitated conversion: in case 1, laparoscopic adrenalectomy was converted to open due to massive bleeding encountered while dissecting the tumor from the splenic hilum and pancreatic tail. The presence of an adrenal capsular hematoma (~20 cm) further complicated dissection, necessitating an open approach to achieve hemostasis and prevent life-threatening hemorrhage.

In case 2, conversion was required due to the friability of the mass and its adherence to surrounding structures, including the splenic hilum and posterior abdominal wall. A Chevron incision was chosen to allow optimal exposure and safe tumor removal.

However, case 1 presented with adhered splenic hilum and tail of the pancreas complicating the intraoperative course requiring subsequent splenectomy which showed features of chronic venous congestion on histopathology. With the advancement and availability of radio-imaging, these cases are being detected and are sometimes associated with large size and adjacent organ adherence which requires a meticulous approach. Comparative analysis of the previously published case reports has been shown in Table 1.

**Table 1:** Overview of previously published case reports

Case	Age/Gender	Presenting symptoms	Vitals	Lab findings	Imaging findings	Diagnosis	Treatment	Outcome
1A: Kumar S et al.^[19]^	40/M	Dyspnea, dizziness, upper abdominal pain	PR: 130, BP: 90/48, RR: 30, Temp: 98.6°F	Hb: 5, WBC: 10,000, Plt: 100 K	USG: 30 × 20 cm mass; CT: 35 × 19 cm fat-density mass with hemorrhage	AML	Laparotomy, right adrenalectomy	Smooth recovery, no recurrence at 6 months
1B: Kumar S et al.^[19]^	50/M (diabetic)	Fever, rigors, upper abdominal pain, vomiting	PR: 130, BP: 86/48, RR: 30, Temp: 103.6°F	Hb: 7.8, WBC: 22,000, Plt: 108 K, Cr: 1.93, BUN: 80	USG: 22 × 17 cm mass; CT: 23 × 21 cm right adrenal mass	Myelolipoma with abscess	Laparotomy, excision, drainage	Smooth recovery, no recurrence at 6 months
2: Ito et al.^[18]^	72/M	Sudden severe left flank pain	BP: 205/108, HR: 85	Hb: 13.5, CRP: 8.58	CT: Left adrenal tumor, fat component, hemorrhage	Myelolipoma with hemorrhage	Embolization, laparoscopic adrenalectomy	Stable post-op
3: Wu Yu et al.^[20]^	59/F	Persistent flank pain, vomiting	BP: 117/86, HR: 76, Temp: 36.9°C	Hb: 13.0, WBC: 8750, D-dimer: 9211	CT: 13.6 × 13.1 cm mass with hematoma	Myelolipoma with hemorrhage	Laparotomy, nephrectomy, aortic repair	CPR, renal impairment, stable follow-up
4: Matsumoto M et al.^[21]^	65/M (diabetic)	Right flank pain, palpable mass	Normal	Hb: 11.4, WBC: 13,600, CRP: 4.0	USG/CT/MRI: 70 × 65 mm fat-rich mass	Suspected ruptured myelolipoma	Conservative	Stable follow-up

The study by Paul et al.^[14]^ highlights the safety of surveillance for AML, even for lesions >4 cm, as long as imaging remains benign and patients remain asymptomatic. Based on this, our follow-up protocol has been refined for optimal monitoring. For residual lesions, we recommend annual radiological surveillance (ultrasound or CT/MRI) to track growth or hemorrhagic changes, with a typical low growth rate of 0.13 cm/year. Giant myelolipomas (≥10 cm) or those with prior rupture may require more frequent follow-up (every 3–6 months for the first year) to monitor recurrence or hemorrhage. Biochemical evaluations should be done periodically to check for adrenal dysfunction. This strategy aligns with the ESE/ENSAT guidelines, emphasizing radiological monitoring and symptom assessment.

## Conclusion

AMLs are rare benign masses detected mostly incidentally and are usually asymptomatic. Few cases present with symptoms due to mass effect. While most cases are small-sized, unilateral, asymptomatic, and non-adhered to other organs, few cases tend to be bilateral and or large-sized. Rarely, it adheres/abutts to surrounding structures, such as the aorta, splenic vessels, tail of pancreas, etc., giving rise to intraoperative complications, a difficult approach. This case series highlights the presence of bilateral AML and possible complications of large-sized AML which mostly causes retroperitoneal hemorrhage and the need for meticulous evaluation, plan and approach.

## Data Availability

Yes the data analyzed during current study are publicly available, available upon reasonable request, or if data sharing is not applicable to this article.

## Research registration unique identifying number (UIN)

Research Registration not done.
